# Predictors of Total Mortality and Echocardiographic Response for Cardiac Resynchronization Therapy: A Cohort Study

**DOI:** 10.5935/abc.20170171

**Published:** 2017-12

**Authors:** Guilherme Ferreira Gazzoni, Matheus Bom Fraga, Andres Di Leoni Ferrari, Pablo da Costa Soliz, Anibal Pires Borges, Eduardo Bartholomay, Carlos Antonio Abunader Kalil, Vanessa Giaretta, Luis Eduardo Paim Rohde

**Affiliations:** 1 Hospital São Lucas da Pontifícia Universidade Católica do Rio Grande do Sul (PUCRS) Porto Alegre, RS - Brazil; 2 Programa de Pós-Graduação em Cardiologia e Ciências Cardiovasculares da Faculdade de Medicina da Universidade Federal do Rio Grande do Sul, Porto Alegre, RS - Brazil

**Keywords:** Heart Failure / mortality, Cardiac Resynchronization Therapy, Stroke Volume, Bundle-Branch Block, Cohort Studies

## Abstract

**Background:**

Clinical studies demonstrate that up to 40% of patients do not respond to cardiac resynchronization therapy (CRT), thus, appropriate patient selection is critical to the success of CRT in heart failure.

**Objective:**

Evaluation of mortality predictors and response to CRT in the Brazilian scenario.

**Methods:**

Retrospective cohort study including patients submitted to CRT in a tertiary hospital in southern Brazil from 2008 to 2014. Survival was assessed through a database of the State Department of Health (RS). Predictors of echocardiographic response were evaluated using Poisson regression. Survival analysis was performed by Cox regression and Kaplan Meyer curves. A two-tailed p value less than 0.05 was considered statistically significant.

**Results:**

A total of 170 patients with an average follow-up of 1011 ± 632 days were included. The total mortality was 30%. The independent predictors of mortality were age (hazard ratio [HR] of 1.05, p = 0.027), previous acute myocardial infarction (AMI) (HR of 2.17, p = 0.049) and chronic obstructive pulmonary disease (COPD) (HR of 3.13, p = 0.015). The percentage of biventricular stimulation at 6 months was identified as protective factor of mortality ([HR] 0.97, p = 0.048). The independent predictors associated with the echocardiographic response were absence of mitral insufficiency, presence of left bundle branch block and percentage of biventricular stimulation.

**Conclusion:**

Mortality in patients submitted to CRT in a tertiary hospital was independently associated with age, presence of COPD and previous AMI. The percentage of biventricular pacing evaluated 6 months after resynchronizer implantation was independently associated with improved survival and echocardiographic response.

## Introduction

Cardiac resynchronization therapy (CRT) has the potential to improve morbidity, mortality, and reverse remodeling in patients with congestive heart failure (CHF) refractory to drug therapy.^1-5^ Over the past few years, based on benefits presented in large clinical trials.^1,4,6^ CRT is being widely used in patients with CHF, decreased left ventricular ejection fraction (LVEF) and presenting prolonged QRS, mainly In the presence of left bundle branch block (LBBB) pattern. In Brazil, there is documentation of an acceptable cost-effectiveness ratio for the use of CRTs in the public system scenario.^7^ However, in addition to the high cost to the health system, approximately 30%-40% of the cases selected for treatment do not benefit from CRT, according to data from large resynchronization studies. For this reason, redoubled efforts should be made in the selection, implantation and follow-up of patients potentially candidates for CRT. The appropriate selection or exclusion of patients with few benefits of therapy is a desired strategy to achieve a higher success rate in CRT.^8^ The evaluation of the responding patients can help in the selection of those who will undergo CRT, making the procedure more cost-effective and avoiding potential adverse events in patients who will not benefit from this therapy.

The main objective of the present study is the evaluation of total mortality and predictors of echocardiographic response to CRT. As secondary objectives, we aimed to evaluate the outcome of total mortality and hospitalization for CHF, survival and functional class of patients after CRT.

## Methods

### Logistics, Inclusion and Exclusion Criteria

This retrospective cohort study included patients with CHF undergoing CRT alone or associated with implantable cardioverter defibrillator (ICD). All patients submitted to the implantation of a multisite cardiac pacemaker (cardiac resynchronizer) by the single health system (SUS), at the Cardiology Department of the São Lucas Hospital of PUC-RS, between 2008 and 2014, were included. Hospital records by checking the list of procedures that included CRT. Patients younger than 18 years of age were excluded from the present protocol, who had only isolated generator exchange or were erroneously allocated on the records (e.g. patients submitted to ICD isolated implantation). Those patients who may have presented technical problems with the pacemaker generator or electrodes were not excluded. The selection of patients was based on 191 patients with CHF and optimized medical therapy (OMT) that were included in the Registry of CRT Implants in the HSL-PUCRS (SUS 2008-2014). After reviewing the medical records, 21 patients were excluded from the CRT registry: 13 patients had undergone a generator change, 4 patients were under 18 years of age, 2 patients had no access to their data, 1 patient was enrolled twice in the registry and 1 patient had been submitted to the implanted ICD isolated.

The study population consists of patients with severe ventricular dysfunction with optimized medical therapy. After completing the inclusion criteria, the patients' data were collected at the Heart Stimulation - Pacemaker outpatient clinic of this institution. The investigator in charge completed a systematized evaluation protocol based on the medical records of these patients until the last outpatient visit. All patients submitted to CRT are followed up at the cardiac stimulation outpatient clinic to evaluate the multisite pacemaker. The first evaluation occurs 30 days after the implant and after every 6 months (or earlier if necessary due to clinical intercurrences). Patients without recent clinical follow-up (last 6 months) were invited to perform a pacemaker review or interviewed by telephone. Mortality data were measured in the database of Death Records of the State Health Department of RS, through the Mortality Information Center (NIS-RS). The follow up of the patients was until 09/22/2015, the final date of the evaluation of the deaths in the Department of Health. A spreadsheet with the data of all the patients participating in the study was cross-referenced with the NIS-RS death chart up to the date established above. In addition, there was an attempt to make a telephone contact with the study participants.

### Clinical outcomes

The main outcomes evaluated were total mortality in the medium term and the echocardiographic response to CRT. The secondary endpoint was the composite of mortality or hospitalization for CHF. The definition of echocardiographic response was considered as an increase in left ventricular ejection fraction (LVEF) ≥ 5% or reduction in final left ventricular systolic volume (LVBSV) ≥ 15%. The definition of response was based on criteria described in previous studies.^9^

### Statistical analysis

Quantitative variables were described as mean and standard deviation, except for the follow-up time, which was presented in median and interquartile range. Qualitative variables were described as absolute and percentage frequency. The distribution of the quantitative variables was evaluated by the Kolmogorov-Smirnov test. In the bivariate analysis, unpaired t-test for quantitative variables and chi-square test for qualitative variables were performed. Fisher's exact test was used when appropriate for qualitative variables.

To evaluate the predictors of echocardiographic response, univariate and multivariate analyzes of Poisson regression with robust variances (binary outcome) were used. Survival analysis was performed by Cox regression and Kaplan Meyer curves. The input criterion for the variable in the multivariate model was that it had a p-value of less than 0.20 in the univariate analysis. Initial sample size of 110 patients was calculated in the WinPEPI program (version 11.43) with the objective of identifying a risk of 2.2 for the ischemic etiology, considering a level of significance of 5%, 80% power and estimated mortality rate of 25%, Based on CARE-HF 10 sub-study data.^10^ The other statistical analyzes were performed in the SPSS version 20.0 program. A two-tailed p value of less than 0.05 was considered statistically significant.

### Ethical aspects

This study consists of a retrospective cohort with the use of patient data, without the identification of included patient data. In this way, the data utilization term was filled out by the researcher responsible. When it was necessary to perform an interview or clinical evaluation with the patient, a free and informed consent form was applied. This research project was approved by the ethics and research committee of Pontifícia Universidade Católica do Rio Grande do Sul (CEP-PUCRS, CAAE: 46267815.3.0000.5336).

## Results

### Patients

We included 170 patients who underwent CRT "again" from 2008 to 2014. The mean follow-up time was 1,011 ± 632 days (median 901 days, interquartile range 489-1473). There were 51 deaths in this period, corresponding to a total mortality rate of 30%. The cardiovascular mortality rate in the period was 15.3%, which corresponds to approximately half of the total mortality. The characteristics of the patients, stratified by mortality and echocardiographic response are described in Tables 1 and 2, respectively.

**Table 1:** Characteristics of the population submitted to CRT stratified by survival

	All patients (n = 170)	Alive (n = 119)	Dead (n = 51)	p value
Idade. Anos Age, years	63.5 ± 12	61.4 ± 11.7	68.3 ± 11.4	p < 0.001^a^
Gender (Male)	115(67.6%)	79(66.4%)	36(70.6%)	p = 0.72^b^
Device type (ICD-CRT)	137(80.6%)	99(83.2%)	38(74.5%)	p = 0.21^b^
Etiology CFH (Non-Ischemic)	89(56.7%)	67(60.4%)	22(47.8%)	p = 0.16^b^
**NYHA Class**				**p = 0.35^b^**
I	1 (0.7%)	1(0.9%)	0(0%)	
II	23 (15.2%)	17(15.7%)	6(14%)	
III	98 (64.9%)	73(67.6%)	25(58.1%)	
IV	29 (19.2%)	17(15.7%)	12(27.9%)	
SAH	131 (79.9%)	90(78.9%)	41(31.3%)	p = 0.83^b^
DM	53 (32.3%)	36(31.6%)	17(34%)	p = 0.86^b^
Prior MI	57 (36.1%)	34(30.9%)	23(47.9%)	p = 0.048^b^
COPD	17 (10.4%)	9(7.9%)	8(16%)	p = 0.16^b^
IRC	40 (24.4%)	24(21.1%)	16(32%)	p = 0.17^b^
Atrial Fibrillation	52(31.7%)	29(25.4%)	23(46%)	p = 0.01^b^
**Medications**				
ACE	97(60.2%)	70(62.5%)	27(55.1%)	p = 0.39^b^
ARA II	34(21.1%)	26(23.2%)	8(16.3%)	p = 0.40^b^
Beta blocker	140(87%)	98(87.5%)	42(85.7%)	p = 0.80^b^
Spironolactone	105(65.2%)	76(67.9%)	29(59.2%)	p = 0.36^b^
**Electrocardiogram**				
QRS	157.6 ± 28.6	156.7 ± 28.5	159.7 ± 29	p = 0.58^a^
LBBB	102(61.8%)	76(66.1%)	26(52%)	p = 0.12^b^
RBBB	10(6.1%)	3(2.6%)	7(14%)	p = 0.009^c^
IBB	10(6.1%)	4(3.5%)	6(12%)	p = 0.069^c^
QRS ≥150ms	90(54.9%)	64(56.1%)	26(52%)	p = 0.73^b^
**Rhythm**				**p = 0.16^b^**
Sinus	111(67.3%)	82(71.9%)	29(56.9%)	
Pacemaker	24(14.5%)	14(12.3%)	10(19.6%)	
Atrial fibrillation	30(18.2%)	18(15.8%)	12(23.5%)	
**Echocardiogram**				
EF	26.8 ± 7	27.8 ± 6.5	24.6 ± 7.5	p = 0.01^a^
LA	4.8 ± 0.7	4.6 ± 0.7	5.2 ± 0.7	p ≤ 0.001^a^
PSAP	44 ± 16.5	40 ± 16.6	50.8 ± 13.7	p = 0.007^a^
LVESV	140 ± 53.1	139.3 ± 50.7	141.8 ± 58.7	p = 0.83^a^
LVDV	202 ± 63	202 ± 57	201.5 ± 74.6	p = 0.97^a^
**Mitral insufficiency**				**p = 0.02^b^**
Minimum	15(17.9%)	14(23.3%)	1(6.7%)	
Light	50 (59.5%)	37(61.7%)	13(54.2%)	
Moderate	14 (16.7%)	6(10%)	8(33.3%)	
**Serious**	**5 (6%)**	**3(5%)**	**2(8.3%)**	
EF post-CRT	34.7 ± 11.4	37.3% ± 11.1	26.9% ± 8.4	p ≤ 0.001^a^
LV electrode (coronary sinus)	158(92.9%)	108(90.8%)	50(98%)	p = 0.11^c^
BP	95.5%(± 9.7)	96.6%(± 8.2)	92.1%(± 12.9)	p = 0.02^a^
BP ≥ 95%	111 (79.3%)	92(86%)	19(57.6%)	p ≤ 0.001^b^

Data expressed as mean ± standard deviation or absolute numbers (percentage).

a Test unpaired;

b Test Chi-square;

c Fisher exact test. ICD -CRT: implantable cardioverter defibrillator + Cardiac Resuscitation Therapy; CHF: congestive heart failure; SAH: systemic arterial hypertension; DM: diabetes mellitus; AMI: acute myocardial infarction; COPD: chronic obstructive pulmonary disease; CRF: chronic renal failure; ACE inhibitor: angiotensin converting enzyme inhibitor; ARA II: angiotensin II receptor antagonist; LBBB: left bundle branch block; RBBB: right bundle branch block; IBB: indeterminate branch block; EF: ejection fraction; LA: left atrium; PSAP: pulmonary artery systolic pressure; LVESV: left ventricular end systolic volume; LVDV: left ventricular diastolic volume; LV electrode: place where electrode was positioned (with percentile positioning electrode via coronary sinus at the side); BP: biventricular pacing at 6 months.

**Table 2:** Characteristics of the population submitted to CRT stratified by the presence of echocardiographic response

	Patients with pre and post implant echo (n = 71)	With ECO Response (n = 42)	No ECO Response (n = 29)	p Value
Idade. anos Age, years	61.6 ± 10.4	61.7 ± 9.9	61.6 ± 11.2	p = 0.97^a^
Gender (Male)	51 (71.8%)	31 (73.8%)	20 (69%)	p = 0.79^b^
Device type (ICD-CRT)	64 (90.1%)	38 (90.5%)	26 (89.7%)	p = 0.9^c^
Etiology CFH (Non-Ischemic)	40 (57.1%)	26 (61.9%)	14 (50%)	p = 0.34^b^
**NYHA Class**				**p = 0.13^b^**
I	0 (0%)	0 (%)	0 (0%)	
II	13 (20.3%)	7 (20%)	6 (20.7%)	
III	38 (59.4%)	24 (68.6%)	14 (48.3%)	
IV	13 (20.3%)	4 (11.4%)	9 (31%)	
SAH	58 (81.7%)	35 (83.3%)	23 (79.3%)	p = 0.76^b^
DM	21 (29.6%)	12 (28.6%)	9 (31%)	p = 1^b^
Prior MI	27 (39.1%)	11 (26.8%)	16 (57.1%)	p = 0.01^b^
COPD	5 (7%)	4 9.5%)	1 (3.4%)	p = 0.32^c^
IRC	18 (25.4%)	10 (23.8%)	8 (27.6%)	p = 0.78^b^
Atrial Fibrillation	17 (23.9%)	10 (23.8%)	7 (24.1%)	p = 1^b^
**Medications**				
ACE	51 (72.9%)	30 (73.2%)	21 (72.4%)	p = 1^b^
ARA II	15 (21.4%)	9 (22%)	6 (20.7%)	p = 1^b^
Beta blocker	63 (90%)	36 (87.8%)	27 (93.1%)	p = 0.7^c^
Spironolactone	48 (68.6%)	28 (68.3%)	20 (69%)	p = 1^b^
**Electrocardiogram**				
QRS	158.4 ± 24.7	162.5 ± 24.4	152.6 ± 24.4	p = 0.13^a^
LBBB	46 (64.8%)	31 (73.8%)	15 (51.7%)	p = 0.08^b^
RBBB	6 (8.6%)	0(0%)	6 (21.4%)	p = 0.002^c^
IBB	6 (8.6%)	4 (9.5%)	2 (7.1%)	p = 0.7^c^
QRS ≥ 150 ms	45 (65.2%)	28 (68.3%)	17 (37.8%)	p = 0.6^b^
**Rhythm**				**p = 0.86^b^**
Sinus	52 (74.3%)	30 (73.2%)	22 (75.9%)	
Pacemaker	9 (12.9%)	5 (12.2%)	4 (13.8%)	
Atrial fibrillation	9 (12.9%)	6 (14.6%)	3 (10.3%)	
**Echocardiogram**				
EF	27.8 ± 7.8	27.8 ± 6.5	24.6 ± 7.6	p = 0.012^a^
LA	4.7 ± 0.8	4.5 ± 0.7	4.9 ± 0.9	p = 0.1^a^
PSAP	43.4 ± 16.2	39.93 ± 16.6	50.81 ± 13.7	p = 0.007^a^
LVESV	143 ± 55.9	155.6 ± 59.2	130.52 ± 49.9	p = 0.08^a^
LVDV	210 ± 62.8	218.5 ± 61.6	201 ± 64	p = 0.28^a^
**Mitral insufficiency**				**p = 0.03^b^**
Minimum	11 (21.2%)	9 (34.6%)	2 (7.7%)	
Light	30 (57%)	15 (57.7%)	15 (57.7%%)	
Moderate	9 (17.3%)	2 (7.7%)	7 (26.9%)	
Serious	2 (3.8%)	0(0%)	2 (7.7%%)	
EF post-CRT	34.4 ± 10.4	39.8% ± 9.4	26.7% ± 6.2	p ≤ 0.001^a^
LV electrode (coronary sinus)	66 (93%)	38 (90.5%)	28 (96.6%)	p = 0.32^c^
BP	95.3 ± 9.3%	98.4 ± 2.6%	90 ± 13.5%	p ≤ 0.001^a^
BP ≥ 95%	48 (77.4%)	36 (92.3%)	12 (52.2%)	p ≤ 0.001^b^

Data expressed as mean ± standard deviation or absolute numbers (percentage).

a Test unpaired;

b Test Chi-square;

c Fisher exact test. ICD-CRT: implantable cardioverter defibrillator + Cardiac Resuscitation Therapy; CHF: congestive heart failure; SAH: systemic arterial hypertension; DM: diabetes mellitus; AMI: acute myocardial infarction; COPD: chronic obstructive pulmonary disease; CRF: chronic renal failure; ACE inhibitor: angiotensin converting enzyme inhibitor; ARA II: angiotensin II receptor antagonist; LBBB: left bundle branch block; RBBB: right bundle branch block; IBB: indeterminate branch block; EF: ejection fraction; LA: left atrium; PSAP: pulmonary artery systolic pressure; LVESV: left ventricular end systolic volume; LVDV: left ventricular diastolic volume; LV electrode: place where electrode was positioned (with percentile positioning electrode via coronary sinus at the side); BP: biventricular pacing at 6 months.

### Total mortality

**Figure 1:**
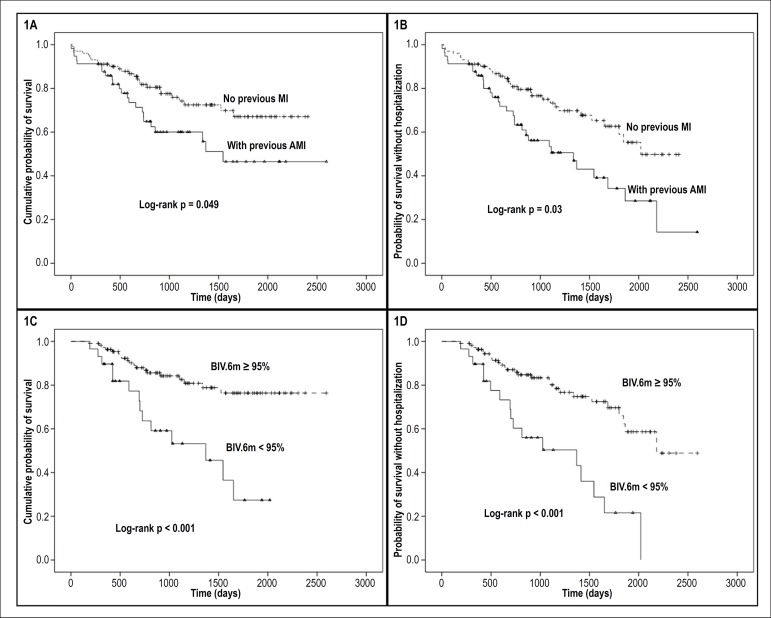
Kaplan-Meyer curve of total mortality (A) and total survival free of death or hospitalization (B) stratified by presence of AMI and total mortality (C) and total survival free of death or hospitalization (D) stratified by the presence of biventricular pacing greater than or equal to 95%.

**Figure 2:**
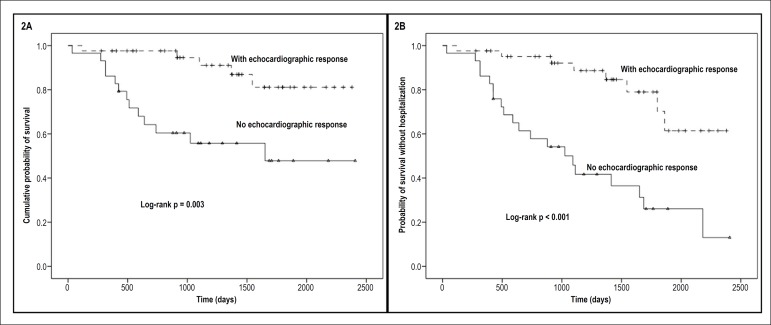
Kaplan-Meyer curve of total mortality (A) and total survival free of death or hospitalization (B) stratified by ecocardiographic response.

Cumulative mortality in the 1st, 2nd and 3rd year of follow-up were, respectively, 11.2% (19 patients), 21.2% (36 patients) and 25.9% (44 patients). Table 3 shows the clinical predictors independently associated with mortality: age (hazard ratio [HR] of 1.05, p = 0.027), chronic obstructive pulmonary disease (COPD) (HR of 3.13, p = 0.015) and prior acute myocardial infarction (AMI) (HR of 2.17; p = 0.049). Age was analyzed as a continuous variable, with an increase in mortality risk of 5% for each additional year of life. As expected, a higher percentage of biventricular stimulation was protective for mortality (HR of 0.972, p = 0.048). For each additional percentage of biventricular stimulation there was a reduction of 2.8% of mortality. In Figures 1 A and 1C, respectively, the survival curve for total mortality can be seen according to the presence of previous AMI and ventricular pacing percentage at the 6-month evaluation. We must highlight the intense effect on mortality in those patients who achieved a biventricular pacing rate greater than 95% in 6 months, with absolute differences of death after 1,500 days of follow-up of approximately 40%, was observed. In addition, we also performed stratification between patients with and without echocardiographic response on total mortality, which can be seen in Figure 2A.

**Table 3:** Univariate analysis and proportional Cox risk model for outcome of total mortality

	Univariate analysis			Multivariate analysis		
	HR	95 % CI	p	HR	95% CI	p
Age	1.05	1.02-1.1	0.001	1.05	1.01-1.09	0.027
COPD	2.33	1.09-5.01	0.030	3.13	1.25-7.82	0.015
Chronic AF	1.79	1.03-3.13	0.039			
LBBB	0.60	0.34-1.04	0.070			
BP.6m	0.97	0.95-0.99	0.008	0.972	0.94-1	0.048
MI prior	1.91	1.08-3.37	0.026	2.17	1.003-4.70	0.049
CRF	1.82	1.005-3.30	0.048			
CRT without ICD	1.63	0.87-3.06	0.130			

HR: "Hazard Ratio"; COPD: chronic obstructive pulmonary disease; AF: atrial fibrillation; LBBB: left bundle branch block; BP.6m: biventricular pacing at 6 months; MI: acute myocardial infarction; CRF: chronic renal failure; CRT: cardiac resynchronization therapy; ICD: implantable cardioverter defibrillator.

### Hospitalization or mortality

During follow-up, 64 patients (37.6%) had death or hospitalization due to CHF. Factors independently associated with mortality or hospitalization for CHF were atrial fibrillation (HR) (HR 2.01, p = 0.03), COPD (HR 2.84, p = 0.01), and prior AMI 2.02, p = 0.03) (Table 4). Similarly, a higher percentage of biventricular pacing was also a protective factor in relation to mortality or hospitalization for CHF (HR 0.97, p = 0.03). In Figures 1B and 1D, respectively, the survival curve can be seen according to the presence of previous AMI and ventricular pacing percentage at the 6-month evaluation.

**Table 4:** Univariate analysis and Cox proportional risk model for outcome of hospitalization or total mortality

	Univariate analysis			Multivariate analysis		
	HR	95 % CI	p	HR	95% CI	p
Chronic AF	1.74	1.05-2.86	0.030	2.01	1.06-3.84	0.03
Age	1.03	1.01-1.06	0.002			
COPD	2.73	1.36-5.45	0.004	2.84	1.27-6.37	0.01
RBBB	3.18	1.56-6.47	0.001			
LBBB	0.51	0.31-0.84	0.008			
BP.6m	0.97	0.95-0.99	0.004	0.97	0.95-0.99	0.035
MI prior	2.08	1.26-3.45	0.004	2.02	1.06-3.87	0.03
QRS > 150	0.65	0.40-1.07	0.095			

HR: "Hazard Ratio"; AF: atrial fibrillation; COPD: chronic obstructive pulmonary disease; RBBB: right bundle branch block; LBBB: left bundle branch block; BP.6m: biventricular pacing at 6 months; MI: acute myocardial infarction.

### Echocardiographic response

According to previously described criteria, 42 patients (59%) presented echocardiographic response. For this analysis, we evaluated only those patients who had echocardiographic data before and after CRT (n = 71). In the multivariate analysis of possible predictors of beneficial echocardiographic response, the independently associated factors were presence of left bundle branch block (LBBB) (HR of 2.58, p = 0.03), higher percentage of biventricular pacing at 6 months 1.12, p = 0.03) and absence of moderate to severe mitral regurgitation (HR of 6.43, p = 0.005), as can be seen in Table 5.

**Table 5:** Univariate and multivariate analysis for echocardiographic response after CRT

	Univariate analysis			Multivariate analysis		
	RR	95 % CI	p	RR	95% CI	p
Minimal Mitral Insufficiency*	4.5	1.24-16.25	0.022	6.43	1.76-23.46	0.005
Mild Mitral Insufficiency	2.75	0.74-10.13	0.128			
COPD	1.39	0.85-2.25	0.184			
Narrowing QRS	1.42	0.84-2.40	0.193			
LBBB	1.53	0.94-2.49	0.085	2.58	1.08-6.17	0.03
BP.6m	3.5	1.27-9.67	0.016	1.12	1.01-1.25	0.030
MI prior	0.57	0.35-0.93	0.026			
Duration QRS	1.01	0.99-1.017				
Left Atrium	0.75	0.55-1.02	0.070			
FLVSV	1.01	1.00-1.01	0.042			
Eject Fraction	0.97	0.95-0.99	0.032			

RR: relative risk; COPD: chronic obstructive pulmonary disease; LBBB: left bundle branch block; BP.6m: bi-ventricular pacing at 6 months; MI: acute myocardial infarction; FLVSV: final left ventricular systolic volume.

* Comparison with moderate to severe mitral regurgitation.

In addition, a stratified survival analysis was performed between patients with and without echocardiographic response (Figure 2). Interestingly, patients who presented an echocardiographic response had a statistically lower risk of total mortality and a high impact on the combined outcome of mortality and hospitalization for HF, with absolute difference greater than 40% after 1,500 days of follow-up.

### Clinical-functional class response

A total of 101 patients were analyzed, data on functional class were available in 12 months. We identified that 71.3% of the patients showed improvement of at least 1 functional class stage in the follow-up of this study. Patients who showed improvement in functional class presented a 69% lower risk of mortality, as assessed by Cox regression (HR of 0.31 for mortality, p = 0.006).

## Discussion

The present study evaluates the effectiveness of CRT in day-to-day practice in a highly complex cardiology center of a university public tertiary hospital in Brazil. Our data demonstrate annual cumulative mortality rate similar to that observed in large international clinical trials, as well as an echocardiographic response rate around 60%. Consistently, we also found that biventricular pacing rate was an important predictor of clinical outcomes. In the national scenario, there are few studies that propose to evaluate the evolution of patients submitted to CRT in the real world, considering the circumstances and peculiarities of the HF care. In this context, our results are important for assessing the effectiveness of CRT at local and national levels, allowing better selection of candidates and better planning of follow-up of these patients. We emphasize that this study was performed only with SUS patients, in a tertiary hospital, which receives patients from all over the state of Rio Grande do Sul for evaluation and treatment. Thus, we believe that this study represents with reliability the reality of the majority of patients submitted to CRT in our country.

Our study included a majority of patients with ICD-CRT implants (80.6%) in relation to CRT alone. This is a retrospective study that evaluated all patients submitted to CRT alone or ICD-CRT in the assessed period. As previously described, there are cost-effectiveness data from the CRT implant in the public scenario.^7^ The CRT implant alone is cost-effective in patients of the Brazilian public system, as demonstrated by Bertoldi EG et al.^11^ This author has shown that for patients eligible for ICD, ICD-CRT implantation is still marginally cost-effective.^11^ We emphasize that all cases were discussed in the Cardiology Department with the participation of the assistant team and the conducts based on the best practices and evidences available at the time of implantation.

Total mortality in this cohort was 30% at a median follow-up of 34 months and 21.2% at 2-year follow-up. We noted a low rate of cardiovascular mortality (15.3%), corresponding to approximately half of the total deaths. These data were derived from the Death Information Service (SIM/RS) of the Health Department of Rio Grande do Sul, which compulsorily incorporates all state death certificates. Mortality of patients submitted to CRT of the CARE-HF study at 1 and 2 years was 9.2% and 18% respectively, with a total mortality of 20% at 29.4 months,^1^ lower than that that we found. Considering 2-year mortality, our data are close to CARE-HF mortality data for this period. Although the overall mortality in our study was greater than that of CARE, cardiovascular mortality in our study was low (51% vs. 83% of cardiovascular mortality in CARE-HF). In a recently published study conducted in a center in Brazil, with patients included between 2008 and 2013, total mortality was 25% (29/116) during follow-up of 34 ± 17 months.^12^

In the present study, independent predictors of total mortality were age, COPD and prior MI. Advanced age and clinical comorbidities are systematically identified as predictors of clinical outcomes in patients with HF.^13-15^ Several previous studies have shown that the patient with HF of ischemic etiology has a worse response to CRT, presumably related to the presence of extensive fibrotic scarring.^16-17^ In spite of this, there are ischemic patients who respond adequately to CRT, and efforts have been employed to identify factors that may identify a greater likelihood of response. The identification of cardiac areas with greater mechanical and/or electrical delay before and during the resynchronizer implantation through echocardiography or through direct measurement can help to refine the indication of CRT in ischemic and non-ischemic patients. Some current studies have already presented preliminary results in the use of these techniques.^3,18-20^ The use of cardiac nuclear magnetic resonance imaging may also help to prevent scarring and may improve the response rate to CRT, particularly in ischemic patients,^21^ with reports of more extensive scarring areas among non-responders.^16,17^

Additionally, we observed that the percentage of biventricular stimulation evaluated at 6 months post-implantation was associated with greater survival. There are consistent data in the literature that point in the same direction. It is recommended that the percentage of biventricular pacing should be as high as possible, ideally close to 100%, which would be associated with a higher probability of clinical improvement.^22^ These findings reinforce the concept that professionals dealing with patients with advanced ICC should be attentive and periodically accompany the patients in the post-implant to obtain the greatest possible clinical benefit. There are specific situations, such as the presence or appearance of atrial fibrillation and ectopies, for example that can significantly decrease the rate of biventricular stimulation, reducing the chance of clinical response. In this study, we can clearly observe that patients with a high percentage of biventricular stimulation (especially when ≥ 95%) had a reduction in total mortality and the combined outcome of death and hospitalization for CHF. Our data suggest that for every additional 1% biventricular stimulation we observed a reduction in mortality risk of 2.8, independent of other factors. This result was observed even considering that the mean biventricular stimulation of our patients was already relatively high. Therefore, our data are in agreement with previous studies and confirm the current concept of "the higher the stimulation the better the response". In addition, there are other potential adjustments related to timing of stimuli in the right and left ventricles and their relation to electrocardiographic findings, which were not possible to be performed in this study because it is a retrospective cohort.

We emphasize that the studied population agrees with the indication criteria in effect at the moment of implantation, since it is an expensive therapy and little available in our environment. Considering that 61% of the patients had LBBB and around 20% of the patients had pacemaker with ventricular pacing and worsening of the functional class, which is also an accurate indication of cardiac resynchronization, approximately 82% of the patients had LBBB or stimulation ventricular pacemaker.^15^

We also evaluated the composite endpoints of mortality and hospitalization for CHF, in addition to echocardiographic response. Regarding the composite endpoint of mortality and hospitalization for CHF, the clinical predictors were AF, COPD and previous MI. Consistent biventricular stimulation was a protective factor. In patients characterized with beneficial echocardiographic response (reverse remodeling) in this study (increase in LVEF and/or reduction in systolic volume), mortality was significantly lower. The main predictors of CRT response, already reported in the literature, are female gender, non-ischemic etiology of CHF, presence of LBBB and QRS ≥ 150 ms.^15,23^ In our study, the percentage of echocardiographic response was 59.1% with 40.9% of non-responders, which would be compatible with the 30-40% range of non-responders reported in the literature. Data from 15 studies grouped in a recent article demonstrated a clinical response rate of 67% and in sub-analysis of the PROSPECT study the echocardiographic response rate was 57%, with 43% of non-responders.^23-26^ The variables independently associated with the echocardiographic response in our analysis were absence of mitral insufficiency, presence of LBBB and biventricular stimulation. We emphasize that the percentage of patients with symptomatic clinical improvement at 12 months, evaluated by NYHA functional class, was 71.3%. Thus, considering the improvement of functional class as a response criterion, we had 28.7% of non-responders to CRT. As in the study by Boidol et al, functional class improvement is significantly correlated with decreased mortality, which gives a higher value to this response criterion, albeit of a subjective nature.^9^

In general, the present study confirms several findings in the literature and may help us to select CRT candidate patients in the national context. It should be noted that after the resynchronizer implantation, close contact should be maintained, with frequent reviews for evaluation and possible adjustments, such as interventions to optimize the percentage of biventricular pacing. These interventions may be medication or through procedures such as AV node ablation in patients with atrial fibrillation, which will allow the percentage of biventricular pacing to be close to 100%.

The main limitations of this study are related to possible information bias, since it is a retrospective review of medical records with a non-ideal rate of nonexistent information. In particular, we had a limited number of patients with adequate echocardiographic data before and after resynchronizer implantation (71), and a missing of 69 patients in the clinical evaluation of the functional class. Many patients did not do any control tests or did them in other different places, at the referral hospital. This is a retrospective real-life study with patients from the national public health system, which makes it relevant in the regional and national context. Limitations in the collection of data from this type of study are a major logistic problem, but we believe that our work can still represent a reliable sample of our population, even though the results obtained are consistent, despite missing data.

## Conclusion

Mortality in patients submitted to CRT at a tertiary hospital in southern Brazil was independently associated with age, presence of COPD and previous MI. The percentage of biventricular stimulation evaluated 6 months after resynchronizer implantation was independently associated with improved survival and lower risk of the combined outcome of death and hospitalization. Adequate echocardiographic response, measured by signs of reverse remodeling, was also associated with a lower risk of total mortality and hospitalization for CHF.
